# Making Evidence Actionable: Interactive Dashboards, Bayes, and Health Care Innovation

**DOI:** 10.5334/egems.300

**Published:** 2019-08-05

**Authors:** Anupa Bir, Nikki Freeman, Robert Chew, Kevin Smith, James Derzon, Timothy Day

**Affiliations:** 1 RTI International, US; 2 University of North Carolina, US; 3 Centers for Medicare and Medicaid Innovation, US

**Keywords:** health care innovation, data science, Bayesian, data visualization, evidence

## Abstract

The results of many large-scale federal or multi-site evaluations are typically compiled into long reports which end up sitting on policymaker’s shelves. Moreover, the information policymakers need from these reports is often buried in the report, may not be remembered, understood, or readily accessible to the policymaker when it is needed. This is not a new challenge for evaluators, and advances in statistical methodology, while they have created greater opportunities for insight, may compound the challenge by creating multiple lenses through which evidence can be viewed. The descriptive evidence from traditional frequentist models, while familiar, are frequently misunderstood, while newer Bayesian methods provide evidence which is intuitive, but less familiar. These methods are complementary but presenting both increases the amount of evidence stakeholders and policymakers may find useful. In response to these challenges, we developed an interactive dashboard that synthesizes quantitative and qualitative data and allows users to access the evidence they want, when they want it, allowing each user a customized, and customizable view into the data collected for one large-scale federal evaluation. This offers the opportunity for policymakers to select the specifics that are most relevant to them at any moment, and also apply their own risk tolerance to the probabilities of various outcomes.

## Introduction

The results of many large-scale federal or multi-site evaluations are typically compiled into long reports which end up sitting on policymakers’ shelves. Moreover, the information policymakers need from these reports is often buried in the report, may not be remembered, understood, or readily accessible to the policymaker when required. The goal of any large-scale evaluation is to produce actionable evidence, making maximal use of the information gathered [1]. This may involve both scans across implementation settings, as well as deep-dives into individual sites’ experiences and outcomes. The challenge is how to synthesize and present this wealth of information to busy policymakers in an engaging and useful manner when they actually need it.

This is not a new challenge for evaluators. While advances in statistical methodology have created greater opportunities for insight, advanced methodology may compound the challenge by creating multiple lenses through which evidence can be viewed. The descriptive evidence from traditional frequentist models, while familiar, are frequently misunderstood, especially with respect to statistical significance [4]. Newer Bayesian methods provide evidence which is intuitive, but less familiar. Bayesian methods incorporate evidence in the form of priors to estimate probability distributions which are predictive of future performance. While these methods are complementary, presenting both increases the amount of evidence stakeholders and policymakers may find useful. In response to these challenges, we developed an interactive dashboard that synthesizes quantitative and qualitative data for a large-scale federal meta-evaluation, allowing users to access a customized view of the evidence they want, when they want it. The dashboard allows policymakers to select features that are most relevant to them at any given moment, while also visually conveying uncertainty in estimates across outcomes.

## Background

In 2012, the Center for Medicare and Medicaid Innovation awarded nearly $1 billion Health Care Innovation Awards to 108 organizations across the country to test innovative ways to deliver health care. The awardees implemented a range of delivery system models. The models included features such as the use of community health workers, health information technology (IT), and workflow redesign, with many of the innovations implementing multiple components (e.g. community health workers and health IT). The Centers for Medicare and Medicaid Services (CMS) contracted with five research organizations to conduct independent evaluations of the awardees’ performance and to file quarterly and annual reports for the three years of performance, beginning July 2012. Each awardee was evaluated separately by an evaluator, referred to as frontline evaluators. Evaluations were coordinated so that common outcomes and measures were used across the awardees.

Our methods for abstracting and synthesizing the information from frontline evaluators using meta-analytic methods have been described elsewhere [2,3]. Briefly, innovation characteristics were coded by trained research staff using a detailed written protocol and structured qualitative coding procedures. With a list of 17 types of health care components, the research staff coded up to 6 types of care provided by each innovation in addition to other innovation characteristics such as whether the targeted population was socially fragile and whether the awardee was a for-profit organization. When possible, frontline evaluators reported effect sizes estimated via difference-in-differences regression models for four core outcomes: total cost of care (TCOC), emergency department (ED) visits, hospitalizations, and hospital readmissions. Total cost of care was measured as dollars per beneficiary per quarter, and the three utilization measures as visits/events per 1000 beneficiaries per quarter. For these estimates, most frontline evaluators used data for beneficiaries touched by the innovation and a comparison group constructed through propensity score methods, and most used data from Medicare or Medicaid claims. We abstracted effect sizes and their standard errors as well as quarterly means and standard errors for each of the four core measures for each awardee from the frontline evaluation quarterly and annual reports. After abstraction, we identified three broad classifications for the innovations: those taking place in the ambulatory-care setting, those taking place in the hospital, and those occurring after an acute care episode. Meta-analysis was undertaken for each of the four core outcomes in each of the three broad settings we identified.

## Design

While we deeply believe that meta-analysis is powerful tool for summarizing evidence, we sought to take the voluminous amount of information from the 135 Health Care Innovation Award interventions generated from our meta-analysis and transform it into actionable, accessible evidence. Our basic premise was that by making evidence interactive and easier to understand, it would promote the use of evidence to inform policy. The tool we developed to do this is an interactive data visualization dashboard. The development of our dashboard was an iterative process informed by an ongoing conversation between analysts, data scientists, and our federal project officer. Guided by the paramount goal of making evidence actionable, we sought to develop a dashboard that satisfied the following four criteria.

First, we wanted our dashboard to integrate both qualitative features of the Health Care Innovation Award innovations with the quantitative results related to the four core measures to truly reflect our mixed-methods approach to analyzing the innovations. Through dialogue among our analysts and our federal project officer, we narrowed down the extensive list of coded features from our review of the innovations into 9 key features that we linked to the four core measures (**Box A**).

**Box A: Highlighted innovation features in dashboard Total cost of care.**

Total cost of care

Innovation setting

Data source

HIT/Telemedicine

Staffing model

Direct care and care coordination

Innovation history

Receipt of a no cost extension

Implementation effectiveness

Second, we wanted to allow policymaking audiences the ability to look both across interventions and deep into particular interventions. This meant the dashboard would need to be dynamic, allowing policymakers to “choose their own adventure” by filtering the visual results to only show the types of innovations that they were interested in understanding better. For example, we wanted our audience to be able to easily filter the visual results to show innovations that resulted in significant cost savings or the ED usage of innovations that used patient navigators. This is a significant departure from how traditional evaluation results in reports are presented. While they are often extensive, the tables and plots tell the narrative of the evaluation through the eyes of the evaluator which may or may not be the relevant story for a policymaker on whom demands for information and decision-making may change over time. Furthermore, we wanted it to be easy for policymakers to find the information they need, to be able toggle quickly and easily between looking closely at a single innovation, looking at a group of innovations with common features, and looking at all of the innovations together. This meant thinking creatively about how this could be done with one-click of the mouse or one drag of a slider.

Third we wanted to improve the interpretability of our results. Traditional frequentist statistics and p-values can be difficult to interpret, as highlighted in the American Statistical Association’s comment on the p-value [4]. To this end, we wanted to include Bayesian estimation techniques to produce estimates that could be interpreted in a probabilistic way. For example, in a frequentist analysis a 95 percent confidence interval for an effect size has the interpretation that 95 percent of the time the method that generated the confidence interval will capture the true effect size. In a Bayesian analysis, the interpretation of a 95 percent credible interval for an effect size has the interpretation that there is a 95 percent chance that the interval contains the effect size. Additionally, using the probabilities generated from a Bayesian analysis enables researchers to answer a class of questions unaddressed in the frequentist setting. Consider the case in which a frequentist analysis infers that the effect of a program is statistically different from zero and that the intervention is beneficial (e.g., a program reduced hospital admissions). A Bayesian analysis can provide further context, answering not only how likely is it that the intervention effect is different from zero, but also how likely it is that the effect is larger than a policy-relevant threshold (e.g., the probability that the program reduced hospital readmissions by 100 or more over a period of time). While the probabilistic interpretations are closer to how people often interpret confidence intervals and want to interpret statistical results, Bayesian methodology is not widely used in health policy evaluation. Due to this unfamiliarity, we included enough information in the dashboard for a user to successfully interpret and discuss the Bayesian estimates provided by the dashboard.

Finally, we wanted our dashboard to be visually appealing and portable. To make it visually appealing, analysts worked closely with a graphic designer, balancing the aesthetics of the visualizations with the amount of information that would be needed to make the dashboard easy to use without extensive instructions. To make it portable, our data scientists developed the dashboard using open source software. This allows the dashboard to be available as a desktop application or hosted on a website. Moreover, the portability makes it ideal for use situations when the data and results included are protected or sensitive and cannot be posted online.

## Methods

### Data

While the data for our meta-evaluation have been described elsewhere [cite], we briefly describe them here. The Centers for Medicare and Medicaid Services (CMS) contracted with five research organizations to conduct independent evaluations of the awardees’ performance and to file quarterly reports over a 4-year period. Each awardee was evaluated separately by an evaluator. Evaluations were coordinated so that common outcomes and measures were used across the awardees. Specifically, for statistical analyses, evaluators used difference-in-differences regression estimates to calculate effect sizes for four core measures: total cost of care (TCOC), emergency department (ED) visits, hospitalizations, and hospital readmissions. Total cost of care was measured as dollars per beneficiary per quarter, and the three utilization measures as visits/events per 1000 beneficiaries per quarter. In addition to statistical analyses, evaluators reported on the qualitative aspects of the innovations and awardees reported details of their innovations to CMS following standardized protocols.

#### Meta-analysis and systematic review

Although 108 organizations were awarded Health Care Innovation Awards, some organization implemented multiple independent innovations, resulting in 135 unique innovations that were separately evaluated. Data for recording or calculating effect size estimates and standard errors for the four core measures for each innovation were abstracted from the evaluators’ quarterly and annual reports. After abstraction, we identified three broad classifications for the innovations: 1) those taking place in the ambulatory-care setting; 2) those taking place in the hospital; and 3) those occurring after an acute care episode.

Using the abstracted effect sizes and standard errors for the four core measures, we synthesized quantitative findings across innovations with a Bayesian random-effects meta-analysis. Unlike a fixed-effect meta-analysis that assumes that the effects of the innovations were the same for all awardees, a random effects meta-analysis models each innovation as having its own effect, with effects coming from a common distribution. In the context of meta-evaluation for delivery system models, a random-effects model captures the inherent heterogeneity between implementing organizations (e.g., differing organizational capabilities or differing approaches to program implementation) as well as other sources of heterogeneity. We used the posterior mean and standard deviation to summarize the meta-analytic effect. Bayesian meta-analysis was used to synthesize effects for the four core measures in each of the three classes of innovations (ambulatory-setting, post-acute setting, and hospital).

Development of the data visualization dashboard occurred through an iterative process between analysts, data scientists, a graphic designer, and the federal project officer directing our effort. The process was guided by the desire to make the dashboard as useful as possible to our policymaking audience, including ease of use and interpretation, without compromising rigor.

## Results

This section describes the various dashboard features with a worked example from the Health Care Innovation Awards meta-evaluation. To help provide context for how the dashboard features interact, Table 1 provides a reference linking the dashboard features to specific Figures.

**Table 1:** Linking dashboard features to specific figures.

Feature	Figure

**Integration of qualitative features and quantitative results**	
Confidence intervals and features side-by-side view	Figure 1
Intervention-specific quarter-by-quarter estimates and features	Figure 3
Credible intervals and features side-by-side view	Figure 4
Intervention-specific effect-size posterior and features	Figure 5
**High-level view**	
Frequentist confidence intervals and program features	Figure 1
Bayesian credible intervals and savings ranges	Figure 4
**Intervention specific view**	
Intervention-specific quarter-by-quarter estimates and features	Figure 3
Intervention-specific effect-size posterior and features	Figure 5
**Frequentist analyses**	
Confidence intervals	Figure 1
Confidence intervals filtered on qualitative feature	Figure 2
Intervention-specific quarter-by-quarter estimates and features	Figure 3
**Bayesian analyses**	
Credible intervals	Figure 4
Intervention-specific effect-size posterior and features, demonstration of probabilistic results	Figure 5
**User-driven views**	
Confidence intervals filtered on qualitative feature	Figure 2
Intervention-specific quarter-by-quarter estimates and features	Figure 3
Credible intervals and features side-by-side view	Figure 4
Intervention-specific effect-size posterior and features	Figure 5

**Figure 1:**
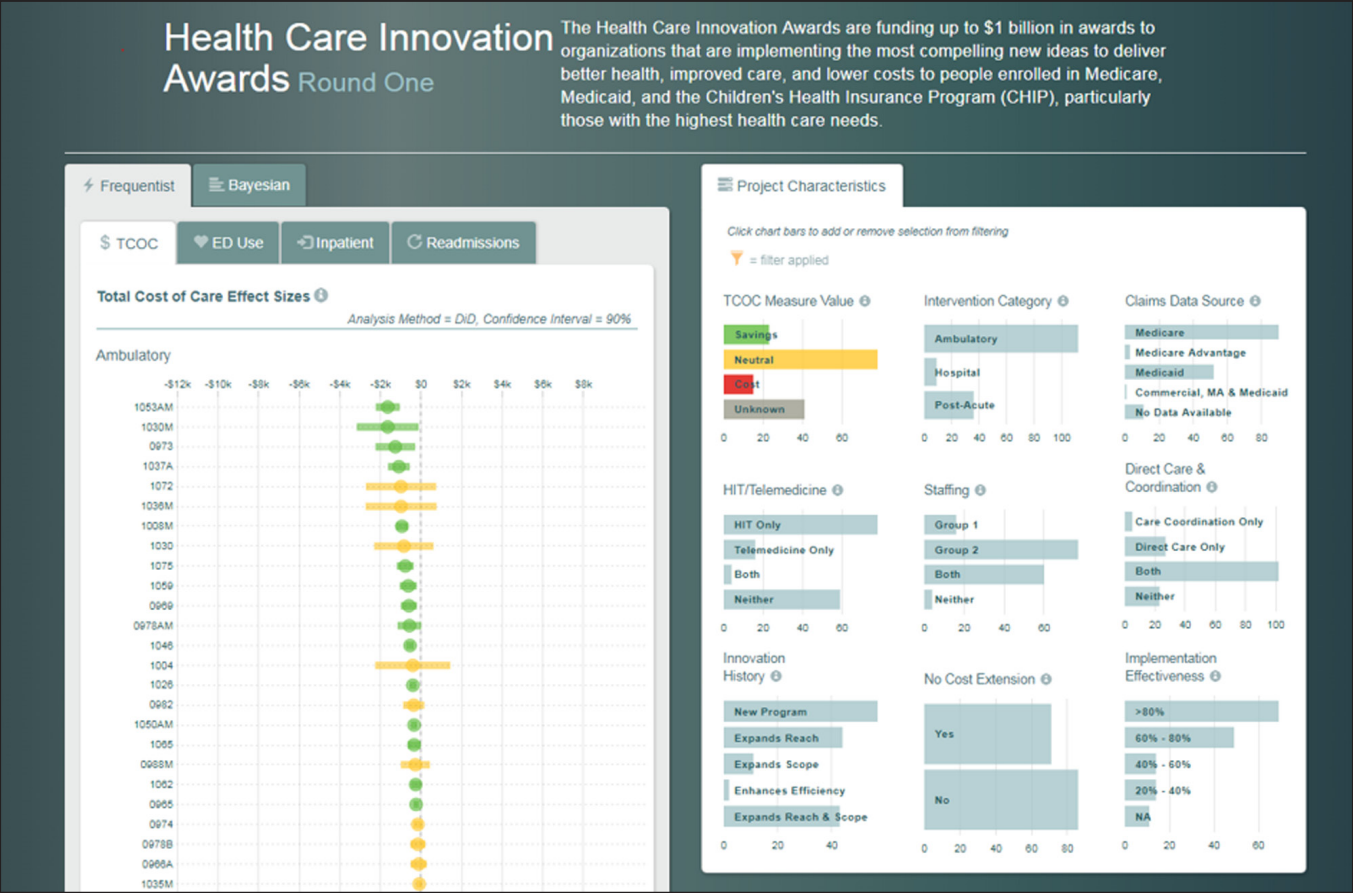
Health Care Innovations Dashboard Screenshot.

Upon opening the dashboard, the view shows a forest plot of a core measure and bar plots of the 9 key innovation features. (Figure 1). Organized by setting (ambulatory care, post-acute, or hospital), effect size estimates and standard errors reported by the evaluators for each innovation are displayed in the forest plot with one forest plot for each of the four core measures on different tabs. The centroids and bars of the forest plot are color coded: green for cost-savings/utilization reduction, yellow for non-statistically significant cost-savings/utilization, and red for cost-increases/utilization increases. Hovering over the centroid and bar for each innovation reveals a box with the exact cost/utilization effect size for the innovation. Adjacent on the page to the forest plot are the bar charts for select project characteristics of interest to CMS staff including intervention setting, claims data source, innovation history, and whether the innovation involved Health Information Technology or telemedicine. Each qualitative feature also includes an information icon which provides a popup describing on how each characteristic is defined.

**Figure 2:**
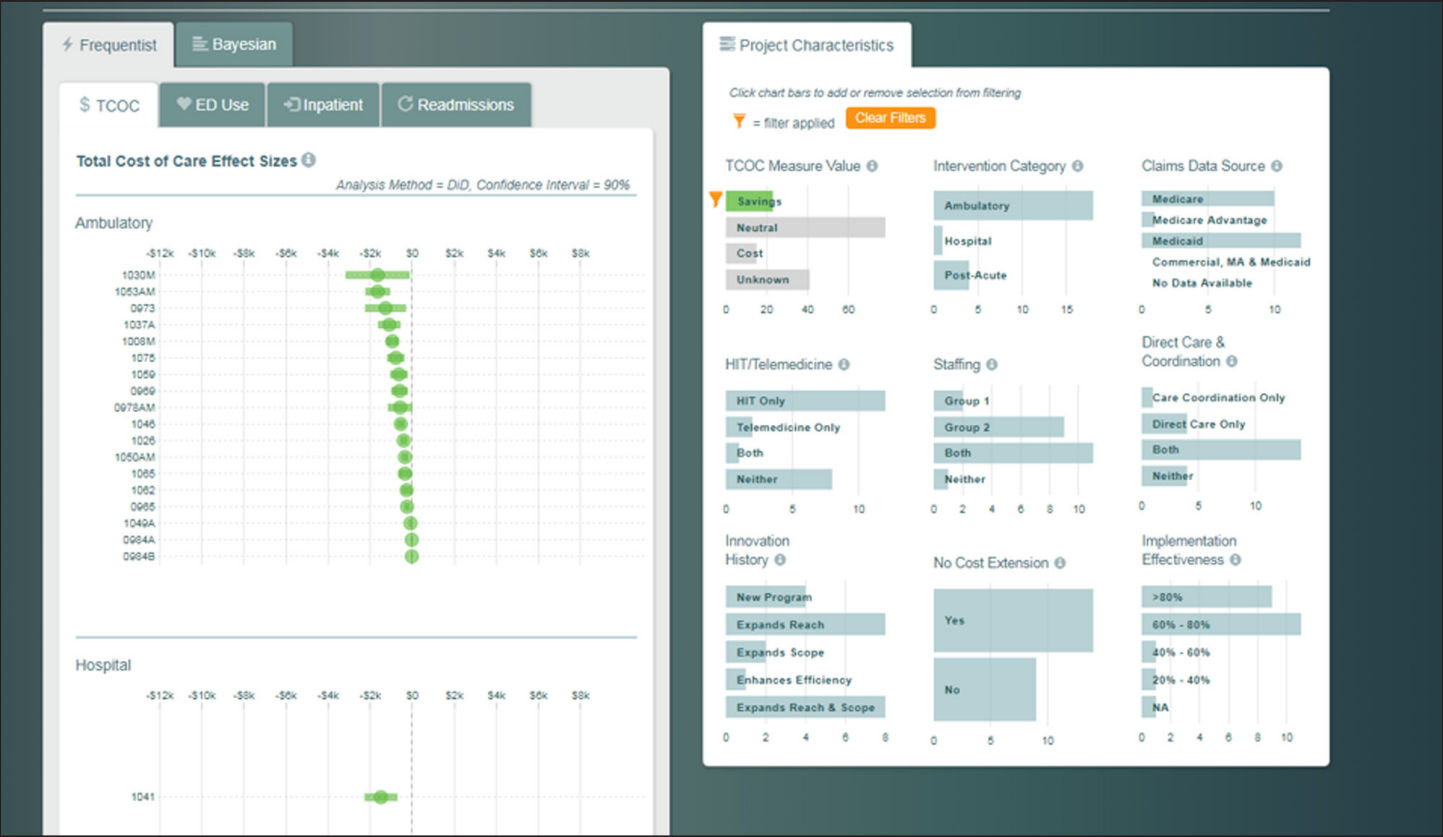
Innovations that Reduced the Total Cost of Care.

From this first view of the dashboard comes the integration of the innovation features and the quantitative evaluation results. For example, one of the bar charts shows the number of innovations that resulted in cost savings, neutral results on savings, losses, and the number that evaluators were not able to estimate an effect for cost. Clicking on the cost savings bar filters the forest plot to show only those interventions with cost savings and updates the other bar charts to reflect characteristics of the cost-savings subset (Figure 2). Results can be filtered further by clicking on other characteristics of interest. This dynamic filtering allows decision makers to easily sort program evaluation results by the characteristic(s) in which they are interested (e.g., programs that showed cost savings, or programs that implemented health IT).

**Figure 3:**
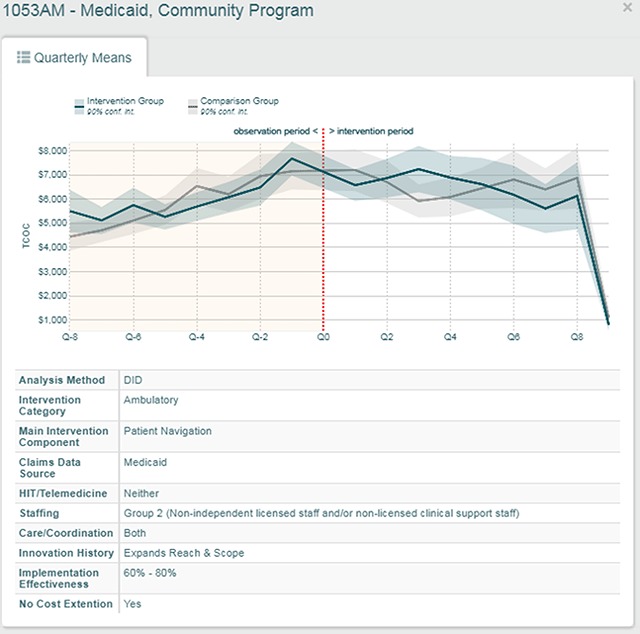
An Example of an Awardees’ Performance on Total Cost of Care over Time.

In addition to being able to look across innovations and filtering on common characteristics for each of the four core measures, the dashboard provides a deep-dive for each innovation. By clicking on an innovation’s bar in the forest plot, a detailed look at innovation specific information pops up (Figure 3). Innovation details include the source of data for the estimate (e.g., Medicare claims data or Medicaid claims data), the primary feature of the innovation (e.g., patient navigation), and the history of the innovation (e.g., whether it was a new innovation or an innovation that expanded either reach or scope). Moreover, the pop-up includes a line chart of quarter-by-quarter mean costs/utilization for the innovation group and comparison group for each innovation.

**Figure 4:**
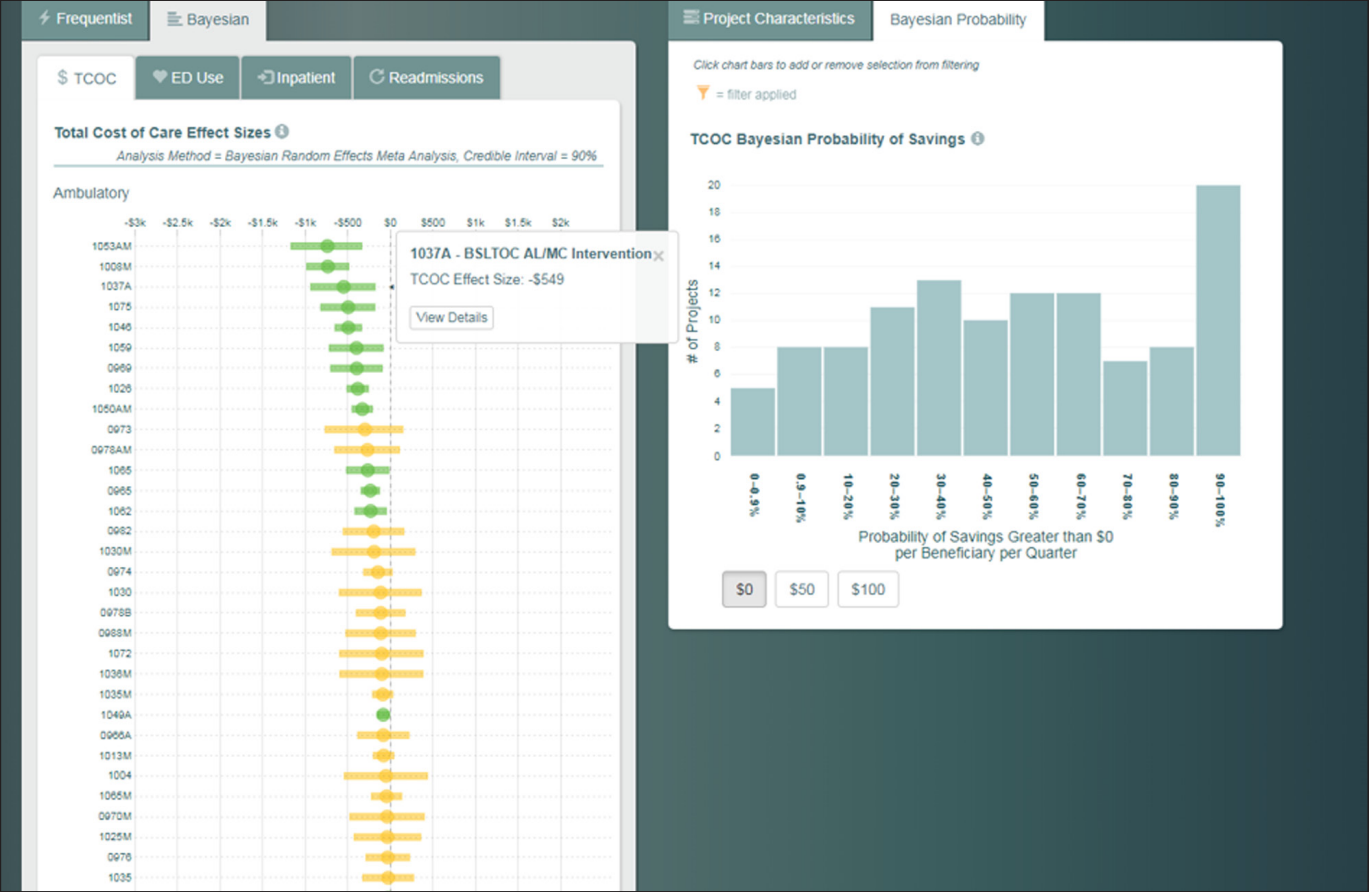
Bayesian Results can be Filtered by Project Characteristics or Probability of Savings.

To this point, we have discussed the display of the program features and the frequentist statistical results for the four core measures reported by the Heath Care Innovation Awards evaluators. Clicking on the “Bayesian” tab at the top of the dashboard transitions to forests plots with Bayesian credible intervals for each innovation. As in the frequentist view, the Bayesian credible intervals can be filtered upon by clicking on the various project characteristics. An extra feature of the Bayesian view is a tab that enables the credible intervals to be filtered on the probability of savings of $0, $50, or $100 per beneficiary per quarter. This is done through a bar chart and a button for each dollar amount (Figure 4). For example, one could click on the $50 button which causes the bar chart to update to show the number of innovations with a 0–0.9 percent probability of $50 of savings per beneficiary per quarter, the number of innovations with 0.9–10 percent probability of $50 of savings per beneficiary per quarter, and so on up to the number of innovations with a 90–100 percent probability of $50 of savings per beneficiary per quarter. In addition to the bar chart updating, dynamic text under the bar chart updates to provide the user the correct interpretation of the display. Moreover, clicking on one or more of the bars filters the forest plot. For example, clicking on multiple bars to identify innovations with 80–100 percent probability of $50 of savings per beneficiary per quarter would update the forest plot to display only innovations with 80–100 percent probabilities of savings.

**Figure 5:**
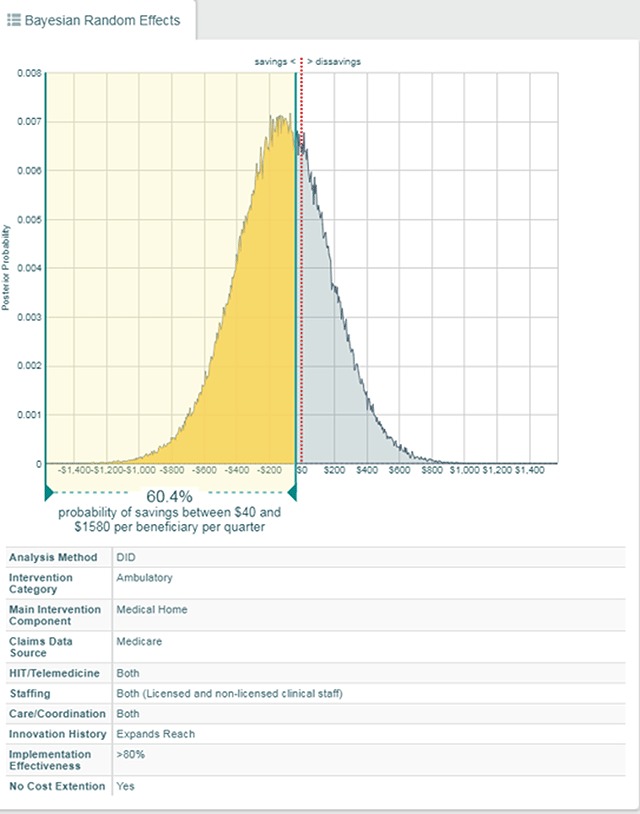
An Example of an Effect Size’s Posterior Density and its Probability of Saving $40–1,580 per Quarter.

In addition to answering questions across innovations using the results from Bayesian meta-analysis, clicking on a single credible interval in the forest plot reveals the posterior density of the estimated effect size (Figure 5). A posterior density is the result of a Bayesian analysis; intuitively, ranges of values with more density (taller density) are more likely than those with lower density (shorter density). The dashboard fits each posterior density with sliders that can be used to highlight different portions of the posterior density. As the user moves the sliders, dynamic text updates to interpret the meaning of the highlighted density. In Figure 5, the sliders have been adjusted to highlight the posterior density left of –$40. In this position, the sliders answer “What is the probability that a particular intervention reduced costs by $40 or more per beneficiary per quarter?” and upon moving the sliders the text updates to answer that the probability is 60.4 percent.

## Discussion

As with any endeavor to synthesize evidence, the meta-evaluation of the Health Care Innovation Awards and development of our dashboard was time and labor-intensive task. It required ongoing coordination and communication within our team and with our federal project officer. However, we believe that generating high quality evidence is only part of the job; its value to policymakers increases when that evidence is easily accessible and can be customized to meet policymakers needs as they arise. Moreover, with our dashboard framework in place we believe that it can be used in a number of settings outside of the Health Care Innovation Awards meta-evaluation. For example, it can accommodate any evaluation in which different sites may have different operating characteristics, whether evaluated individually or jointly (e.g., different hospitals in a single state or states in a multistate test of a health delivery system model).

In addition to using our dashboard in new settings, we also hope to expand the capabilities of our dashboard. First, for the Health Care Innovation Awards meta-evaluation, the focus was on answering, “did the innovations work and by how much?” However, we know that the decisions of policymakers cannot always wait until the end of the evaluation. The natural next step for the dashboard is to handle multi-stage decision problems by adding a temporal element for “online” decision making. Analytically, this would involve adding a predictive component, such as Bayesian random forests or support vector machines to aid policymakers in making interim assessments, decisions, and projections for the future. Moreover, the current dashboard does not present any explicit modeling of the patterns associated between the program features and the four core outcomes. All associations are based on filtering on the key features. We believe that explicit modeling and visualization of the relationships would be an important improvement in extracting the most information available from the data. For example, in the meta-evaluation context, Bayesian network modeling could be an intuitive and powerful tool for intuitively and probabilistically describing the relationship between program features and outcomes. In the case with individual-level data, Bayesian neural networks could provide a powerful, flexible model.

## Conclusion

The dashboard for the meta-evaluation of the Health Care Innovation Awards is a user-friendly, interactive display for making evidence actionable for policymakers. Guided by a desire to make our results as interpretable and relevant as possible, we created a dashboard to inform policymakers on whether to scale-up, continue, or end an ongoing innovation. Making it easier to understand evidence is of course not a guarantee that it will be used in policymaking, but it should improve the likelihood relative to evidence that is more challenging to understand.
